# Interventions and turfs

**DOI:** 10.4103/0971-3026.54872

**Published:** 2009-08

**Authors:** Bhavin Jankharia

**Affiliations:** Editor-in-Chief, The Indian Journal of Radiology and Imaging, ‘F’, Bhaveshwar Vihar, 383, Sardar V P Road, Mumbai - 400 004, India. E-mail: editor@ijri.com

The definition of “interventional radiology” (IR) has always been a bit hazy, leading to situations where someone in the past even considered calling a “barium enema” an interventional procedure.[1] Nevertheless, in the popular radiology realm, it is the glamorous face of radiology, the interventional radiologists appearing as swashbuckling catheter-swishers, charging forth into uncharted regions of the patient, to diagnose and treat.

A rigid definition of IR would probably mean “someone who uses image guidance to treat.” But, this negates the efforts of many, many radiologists who still “intervene” into patients' bodies, but are not considered to be similar to the glamorous vascular interventionalists.

This month's issue has many articles on this subject, ranging from the use of PET to guide biopsies,[2] to the use of an automated system to help in biopsying difficult areas of the body[2] and, finally, the use of ethanol in treating thyroid cysts.[3] The first two articles mentioned above discuss only diagnostic procedures but nevertheless represent a growing part of IR. In fact, if IR as a discipline were to broaden its base to include those who perform guided biopsies as an integral part of the specialty, many of the problems of this subspecialty might perhaps get solved.

We also feature an article that one reviewer had categorically rejected saying that the subject matter was not relevant to a radiology journal. This article titled “Impact of USG on central venous catheter insertion in intensive care”[4] talks about the use of ultrasonography in guiding the placement of central venous catheters, which otherwise are usually inserted using a relatively blind landmark technique. Even though the vast majority of these procedures are not performed by radiologists, an article such as this is an eye-opener for us, showing us how imaging is being used in other fields, especially in the intensive care unit, where non-radiologists are by and large taking care of their own radiology needs, bypassing us completely. Articles such as these should serve as wake-up calls.

From this issue onwards, we are also starting a series of review articles on various aspects of tuberculosis. This issue features an article on “musculoskeletal tuberculosis,” written by Dr. Hoenacker and his colleagues.

As we enter the last quarter of 2009, it is heartening to note that three years have passed by with a significant change in all aspects of our journal and we hope that this continues into the future.
